# Ocular Rigidity and Outflow Facility in Nonproliferative Diabetic Retinopathy

**DOI:** 10.1155/2015/141598

**Published:** 2015-04-14

**Authors:** Theonitsa Panagiotoglou, Miltiadis Tsilimbaris, Harilaos Ginis, Nikos Karyotakis, Vaggelis Georgiou, Pavlos Koutentakis, Ioannis Pallikaris

**Affiliations:** ^1^Department of Ophthalmology, University Hospital of Heraklion, 71110 Heraklion, Greece; ^2^Department of Ophthalmology, Venizeleio General Hospital of Heraklion, 71409 Heraklion, Greece; ^3^Institute of Vision and Optics, School of Medicine, University of Crete, 71110 Heraklion, Greece; ^4^Department of Social Medicine, School of Medicine, University of Crete, 71110 Heraklion, Greece

## Abstract

*Purpose.* To compare ocular rigidity (OR) and outflow facility (C) in patients with nonproliferative diabetic retinopathy (NPDR) and control subjects. *Methods*. Twenty-four patients with NPDR (NPDR group) and 24 controls (control group) undergoing cataract surgery were enrolled. NPDR group was further divided into patients with mild NPDR (NPDR1-group) and patients with moderate and/or severe NPDR (NPDR2-group). After cannulation of the anterior chamber, a computer-controlled device was used to infuse saline and increase the intraocular pressure (IOP) in a stepping procedure from 15 to 40 mmHg. Ocular rigidity and outflow facility coefficients were estimated from IOP and volume recordings. *Results*. Ocular rigidity was 0.0205 *μ*L^−1^ in NPDR group and 0.0202 *μ*L^−1^ in control group (*P* = 0.942). In NPDR1-group, OR was 0.017 *μ*L^−1^ and in NPDR2-group it was 0.025 *μ*L^−1^ (*P* = 0.192). Outflow facility was 0.120 *μ*L/min/mmHg in NPDR-group compared to 0.153 *μ*L/min/mmHg in the control group at an IOP of 35 mmHg (*P* = 0.151). There was no difference in C between NPDR1-group and NPDR2-group (*P* = 0.709). *Conclusions*. No statistically significant differences in ocular rigidity and outflow facility could be documented between diabetic patients and controls. No difference in OR and C was detected between mild NPDR and severe NPDR.

## 1. Introduction

Diabetes mellitus is increasing at an alarming rate. In 1997, 124 million people worldwide had diabetes, with 97% of them having type 2 diabetes [1]. By the year 2012, the total number of people with diabetes was more than 371 million [2]. The development of diabetic complications is a major cause of morbidity and mortality and is an ever-increasing burden to healthcare authorities in both developed and developing nations [2]. Diabetic retinopathy, one of the major diabetic complications, may lead to visual disability and blindness.

Diabetes is characterized by high blood glucose concentrations that lead to increased production of free radical intermediates [3–9]. The resulting glycative, glycoxidative, carbonyl, and oxidative stress play a key role in pathogenesis of diabetes. Glycation is considered the cause of many of the damaging late complications. Initially, glycation affects the interactions of collagen with cells and other matrix components, but the most damaging effects are caused by the formation of glucose-mediated intermolecular cross-links. These cross-links decrease the critical flexibility and permeability of the tissues and reduce turnover [10, 11].

Ocular rigidity is a macroscopic parameter characterizing the relationship between pressure and volume changes in the human eye. Ocular rigidity depends on the architecture and material properties of the eye globe. Measurements of OR have mainly been performed by means of paired Schiotz tonometry [12] or invasive manometric devices [13–18]. These measurements pertain to the injection (or displacement) of a given volume in the eye and measurement of the associated intraocular pressure change. Ocular volume, age, intraocular pressure, axial length, arterial pressure, and ocular blood volume have all been suggested to influence ocular rigidity [12, 14, 15]. Moreover, rigidity has been shown to be altered in patients with age-related macular degeneration [13] and glaucoma [16]. In Friedenwald's studies, ocular rigidity was found to be altered in uveitis patients [12]. Patients with alterations in biomechanical properties of the cornea, such as keratoconus or osteogenesis imperfecta, exhibit lower ocular rigidity coefficient compared with normal controls; corneal thinning was found to correlate with ocular rigidity in these patients [19–21]. So far, little is known about the possible relationship between ocular rigidity and diabetic retinopathy. Outflow facility is a measure of the resistance of the conventional outflow pathway of the aqueous humor. A former study has shown that outflow facility coefficient is low in diabetic patients compared to normal population [22].

Although it is well known that diabetes mellitus interferes with the vascular integrity of the ocular tissues [23], the relationship between diabetes and ocular rigidity has received little attention. The purpose of the present study is to investigate whether OR is modified in diabetic patients compared to normal controls and evaluate a possible correlation of OR with the severity of diabetic retinopathy.

## 2. Materials and Methods

### 2.1. Patients

Patients scheduled for cataract surgery were recruited from the Ophthalmology Department, Venizeleio General Hospital of Heraklion. The study protocol was carried out after the approval from the Venizeleio General Hospital Institutional Review Board. The study was in accordance with the Declaration of Helsinki; a written informed consent was obtained from all participants.

Twenty-four patients diagnosed with mild, moderate, and severe NPDR and 24 controls matched for axial length were included in the study. One eye per patient was included in the study. Patients with normal eye exams (except for cataracts), IOP less than 22 mmHg in at least two visits, and no history of diabetes were recruited and were eligible to be included in the control group. All diabetic participants had late onset diabetes. Patients with medically treated systemic hypertension, without findings of hypertensive retinopathy in dilated fundoscopy, were considered eligible to be included in the study. Exclusion criteria for both diabetic patients and controls were the presence of ophthalmic disease other than cataract and nonproliferative diabetic retinopathy (for study patients), history of previous intraocular surgery, laser treatment or ocular trauma, and history of severe cardiovascular or pulmonary disease. Moreover, patients with connective tissue disorder, hereditary or autoimmune, were excluded from the study, due to possible alteration of scleral elasticity.

All patients underwent a thorough ophthalmic assessment including slit lamp biomicroscopy, Goldmann applanation tonometry, and dilated fundoscopy. Axial length and central corneal thickness (CCT) measurements were performed with ultrasonic biometry and pachymetry (Ocuscan, Alcon Laboratories Inc., Irvine, CA).

Patients with mild, moderate, or severe NPDR attending the fundus outpatient clinic were recruited in the NPDR group. The diagnosis of NPDR was based on the appearance of the retina on dilated fundus examination. NPDR was classified to mild, moderate, or severe according to the Early Treatment Diabetic Retinopathy Study Classification [24].

### 2.2. Measurement Procedure

**Figure 1:**
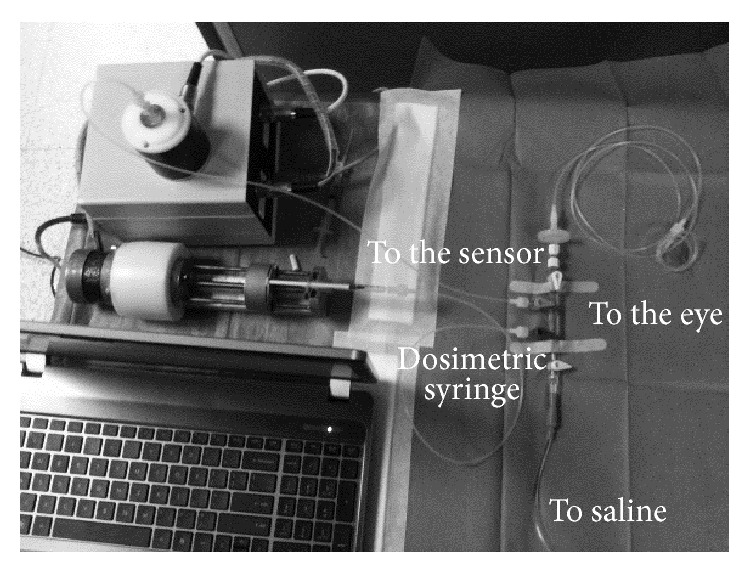
Setup of the system for the intraoperative measurement of ocular rigidity and outflow facility.

The measurement procedure has been explained in detail before [14, 15, 17]. Briefly, a custom computer-controlled device for the measurement and control of IOP was employed. This device consists of a pressure sensor (sampling rate 200 Hz, effective pressure sensitivity 0.05 mmHg), a dosimetric syringe drive unit (volume sensitivity 0.08 *μ*L per step), and a circuit of sterile inextensible tubes (Vygon, Ecouen, France), filled with saline solution (Figure 1). Custom software was used for system control and data acquisition (LabView; National Instruments Inc., Austin, TX).

Pupil dilation was performed with phenylephrine 5%, tropicamide 1.0% drops. Topical anesthesia was applied with proparacaine hydrochloride drops and the measurement was performed under sterile conditions in the operating theatre, before cataract surgery. A standard procedure was followed during both the preparation of the system and the measurement in order to minimize the possibility of leaks or trapped air in the system. The whole measurement was performed under the operating microscope.

In every measurement, after system calibration, the anterior chamber was cannulated with a 21-gauge needle attached to the end of the tubing circuit, in order to establish free communication between the eye and the measurement device. The IOP was then set to the level of 10 mmHg with appropriate saline solution and aqueous humor exchange and was increased from 10 to 40 mmHg, by injecting microdoses of saline solution in the eye in a stepping procedure in all eyes tested. After each 4 *μ*L infusion step, a 2-second real-time continuous recording of IOP was acquired, in order to record the rhythmic IOP oscillations with the heartbeat. When the IOP reached 40 mmHg, the infusion stopped, and the sensor continuously recorded the decreasing IOP for a period of 4 minutes. The relationship between IOP increase and infused volume was used to calculate the ocular rigidity coefficient [17] while the IOP decay during outflow was used to calculate the outflow facility coefficient [18].

### 2.3. Data Analysis and Statistics

Normality of distribution was assessed with the Shapiro-Wilk test for each variable. Parameters are presented as either mean ± SD for continuous variables or frequencies (*N*) and percentages (%). Bivariate comparisons were performed with nonparametric Mann-Whitney test for continuous variables and Pearson's *χ* ^2^ or Fisher exact test when less than 5 subjects were expected in a cell, for categorical variables.

Correlation analysis was performed with Spearman's rho correlation coefficient, to estimate the strength of the association between two continuous variables.

All hypothesis testing was conducted assuming a 0.05 significance level and a two-sided alternative hypothesis. Data analysis was performed by using SPSS version 17 statistical software (SPSS, Inc., Chicago, IL).

## 3. Results

Twenty-four patients with nonproliferative diabetic retinopathy (NPDR group) and 24 controls (control group) undergoing cataract operation were enrolled. NPDR group was further divided into 14 patients with mild NPDR (NPDR1-group) and 10 patients with moderate and severe NPDR (NPDR2-group).

Mean age was 71.71 ± 8.0 years in the NPDR group and 73.00 ± 11.1 in the control group (*P* = 0.278). There were 11 females in NPDR group and 16 females in control group (*P* = 0.146). There was no difference in axial length between two groups (NPDR group 23.13 ± 0.85 mm and control group 23.13 ± 0.81 mm, *P* = 0.96). Central corneal thickness (CCT) was 550 ± 32 *μ*m in NPDR group and 541 ± 42 *μ*m in control group (*P* = 0.564). In Table 1, age, gender, axial length, CCT, and preoperative IOP are compared between NPDR group and control group (left) and between NPDR1-group and NPDR2-group. There was no difference in sex, systemic hemodynamics, or preoperative IOP between patients and controls. There were no complications during or after the operation related to the measurement procedure.

**Figure 2:**
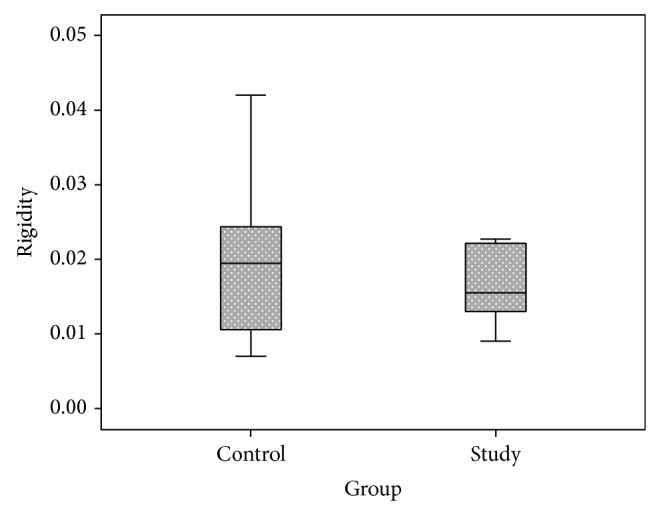
Ocular rigidity (*μ*L^−1^). Boxplots of mean ocular rigidity coefficient in NPDR group (*n* = 24) and control group (*n* = 24).

**Figure 3:**
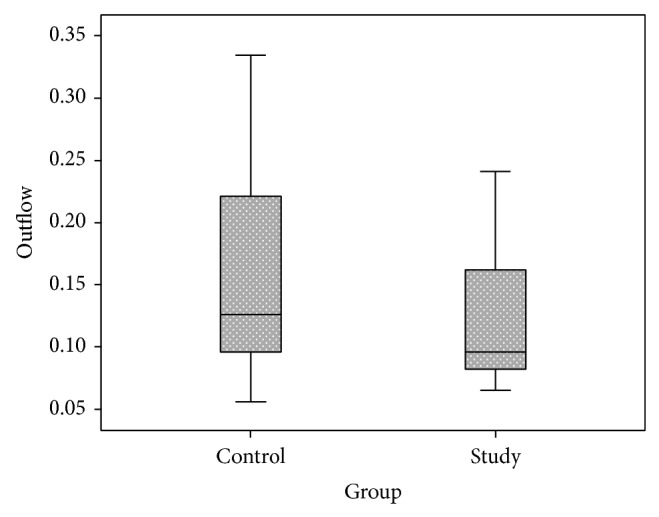
Outflow facility (*μ*L/min/mmHg). Boxplots of mean outflow facility coefficient in NPDR group (*n* = 24) and control group (*n* = 24).

The OR coefficient was 0.0205 ± 0.125 *μ*L^−1^ in NPDR group and 0.0202 ± 0.0118 *μ*L^−1^ in control group (*P* = 0.942). In NPDR1-group OR was 0.017 ± 0.009 and in NPDR2-group it was 0.025 ± 0.015 *μ*L^−1^ (*P* = 0.192) (Figure 2). C was 0.120 ± 0.048 *μ*L/min/mmHg in NPDR group compared to 0.153 ± 0.078 *μ*L/min/mmHg in the control group at an IOP of 35 mmHg (*P* = 0.151) (Figure 3). There was no difference in C between NPDR1-group and NPDR2-group at the same IOP (0.119 ± 0.048 and 0.120 ± 0.059, resp., *P* = 0.709).

## 4. Discussion

Ocular rigidity, as described by Friedenwald, is a measure of the resistance that the eye exerts to distending forces [12]. This parameter describes the elasticity of the ocular shell, especially the sclera and cornea and the compressibility of the choroid, assuming that the other ocular compartments are practically incompressible. There has been considerable interest in the impact of ocular rigidity on ocular diseases, such as glaucoma, age-related macular degeneration, myopia, uveitis, keratoconus, and diabetes [12–17, 19–21].

Diabetes mellitus is known to affect biomechanical properties of the tissues [25]. High glucose concentrations lead,* via *several mechanisms (the polyol pathway, hexosamine pathway, advanced glycation end products (AGEs) pathway, and protein kinase C), to increased production of free radical intermediates. The effects of AGEs are twofold: mechanical effects are due to intermolecular cross-linking that lead to major alterations of the physical properties of tissues (e.g., increased fibril stiffness). Moreover, AGEs alter the biochemical profile of the collagen molecule and if they occur at specific sites they can affect the intermolecular and cell-collagen interactions [8–11]. Since AGEs influence collagen molecule and collagen interactions, collagen in sclera and cornea in diabetes could have sustained mechanical and biochemical alterations leading to changes in ocular rigidity. The findings of Şahin et al. [26], showing that diabetes affects corneal biomechanics and results in lower corneal hysteresis values than those in healthy control subjects, are in accordance with this hypothesis. In addition, increased AGE accumulation distributed around blood vessels has been found in the retinal vessels of diabetics, increasing with the severity of retinopathy [27–29]. Based on the common background of AGE accumulation in retinal perivascular space and sclera, we hypothesized that possible changes in ocular rigidity in diabetes may evolve in parallel with diabetic retinopathy.

In this study, we did not detect a statistically significant difference of OR value between diabetic patients and controls. A clear trend for lower OR was documented in patients with mild NPDR compared to patients with moderate and severe NPDR, although the difference did not reach statistical significance. Arora and Prasad estimated scleral rigidity according to modified Friedenwald monogram and outflow facility with tonography in diabetic patients. They reported that there were no statistically significant differences in ocular rigidity measurements between patients with nonproliferative diabetic retinopathy compared with age-matched control patients [20]. Although the methodology used by these investigators can be considered indirect compared to the direct manometric technique utilized in our study, the findings of their work are similar to ours.

A trend for lower outflow facility at an IOP of 35 mmHg of diabetic patients compared to controls was found in our study. This difference did not reach a statistical significance. Arora and Prasad found lower values of C in patients with NPDR compared to controls and diabetic patients with PDR [22]. We were not able to confirm the correlation of C with PDR since no patients with proliferative diabetic retinopathy were enrolled in our study. Larsson et al. have found that the dynamics of aqueous humor are not affected to any clinically significant extent in the early or middle stages of diabetic retinopathy [30]. Outflow facility was not significantly different in diabetics compared to control subjects. Again, although the methodology of these investigators was indirect compared to the direct manometric technique used in our study, they, similarly to us, could not find significant correlation between tonographic facility of outflow and the severity of retinopathy. However, it is important to recognize that our outflow facility measurements suffer a significant limitation, since C was measured after pupil dilatation. Both trabecular meshwork and uveoscleral outflow are known to be affected by cycloplegic medications [31]. We are currently working on the development of an accurate noninvasive device that may permit measuring both ocular rigidity and outflow facility without mydriasis [32].

One of the factors that influence the OR coefficient is age [12, 13, 15]. An increase in ocular rigidity coefficient with increasing age was first reported by Friedenwald in a large series of human eyes [12]. An increase in stiffness and a decrease in thickness of the peripapillary and posterior sclera with age have also been reported in primates [33], while measurements in human scleral segments also indicate a relationship between age and elasticity of the sclera [34]. A decrease in C with age has also been reported in some [35] but not all studies [36]. The relationship between age and ocular rigidity is of importance as it may underlie the susceptibility to age-related ocular disease [37]. In our study, patients in all groups were older than 70 years and there was no statistically significant difference in age between diabetic patients and the control group, limiting this way the possibility for an age-related bias.

In conclusion, in this study we were not able to document any significant difference in ocular rigidity and outflow facility between diabetic patients and normal controls. Diabetic patients exhibit a trend for higher ocular rigidity values as the severity of diabetic retinopathy worsens. We also found a trend for lower outflow facility in patients with NPDR compared to controls. Additional work is necessary in order to elucidate whether there is a correlation between ocular rigidity and diabetic retinopathy. To the best of our knowledge, this is the first study to investigate the in vivo ocular rigidity and outflow facility in diabetic patients with a manometric method. Significant limitations of our work include the relatively small number of patients enrolled, the absence of a group of patients with proliferative diabetic retinopathy, and the pupil dilation during outflow facility measurement. However, our data can serve as a point of reference for future larger scale studies.

## Figures and Tables

**(a):** 

	Group		*P* value^∗^
	Control (*n* = 24)	Study (*n* = 24)	
Age (years), mean ± SD	73.00 ± 11.1	71.71 ± 8.0	0.278
Gender, *N* (%)			
Male	8 (33.3)	13 (54.2)	0.146
Female	16 (66.7)	11 (45.8)	
Eye, *N* (%)			
Right	8 (33.3)	13 (54.2)	0.146
Left	16 (66.7)	11 (45.8)	
Axial length (mm), mean ± SD	23.13 ± 0.8	23.13 ± 0.8	0.959
CCT (*μ*m), mean ± SD	540.79 ± 41.6	549.67 ± 32.3	0.564
Preoperative IOP (mmHg), mean ± SD	13.79 ± 2.4	14.48 ± 2.9	0.387

^∗^Statistically significant differences at *P* < 0.05, based on Mann-Whitney *U* test for two independent samples and chi-square of Pearson test.

**(b):** 

	Study group		*P* value^∗^
	NPDR1 (*n* = 14)	NPDR2 (*n* = 10)	
Age (years), mean ± SD	73.60 ± 4.7	70.35 ± 9.7	0.437
Gender, *N* (%)			
Male	8 (57.1)	5 (50.0)	0.146
Female	6 (42.9)	5 (50.0)	
Eye, *N* (%)			
Right	7 (50.0)	6 (60.0)	0.697
Left	7 (50.0)	4 (40.0)	
Axial length (mm), mean ± SD	23.20 ± 1.0	23.07 ± 0.7	0.709
CCT (*μ*m), mean ± SD	551.00 ± 28.0	548.71 ± 36.1	0.796
Preoperative IOP (mmHg), mean ± SD	14.50 ± 3.2	14.46 ± 2.8	0.976

^∗^Statistically significant differences at *P* < 0.05, based on Mann-Whitney *U* test for two independent samples and chi-square of Pearson or Fisher exact test.
